# Continuous Effectiveness of Pneumococcal 13-Valent Conjugate Vaccine on Pediatric Pneumococcal Otomastoiditis: Results of 15 Years of Active/Prospective Surveillance in a Mexican Hospital on the Mexico-US Border

**DOI:** 10.7759/cureus.17608

**Published:** 2021-08-31

**Authors:** Enrique Chacon-Cruz, Erika Z Lopatynsky

**Affiliations:** 1 Pediatric Infectious Diseases, Tijuana General Hospital, Tijuana, MEX; 2 Family Medicine and Public Health, University of California San Diego, San Diego, USA

**Keywords:** otomastoiditis, pneumococcal otomastoiditis, pneumococcal conjugate vaccine, vaccine effectiveness, children

## Abstract

Introduction

The effectiveness of the 13-valent pneumococcal conjugate vaccine (PCV13) on sepsis, meningitis, pneumonia, and even acute otitis media has been proved in many studies. Nonetheless, the impact of PCV13 on otomastoiditis (OM) in children has barely been reviewed. In the past, we published a 13 years pneumococcal OM study from our hospital. This is a continuation of our active surveillance and is the first Latin American, prospective study examining the effectiveness of this vaccine on pneumococcal pediatric OM.

Methods

Active surveillance identifying patients < 16 years of age with OM admitted at the “Hospital General de Tijuana” was performed from October 1, 2005, to September 30, 2019. Diagnosis of OM was based on clinical exam (postauricular tenderness, erythema, and swelling causing protrusion of the auricle) and computerized tomographic signs (opacification of the mastoid air cells and middle ear). We used either conventional culturing or PCR to isolate bacterial pathogens, while to further *Streptococcus pneumoniae* serotype identification we used the Quellung Reaction (Statens Serum Institute^®^) or PCR. To assess pneumococcal conjugate vaccines effectiveness (VE), we counted cases per month before any pneumococcal conjugate vaccine was implemented (19 months surveillance), during the 7-valent pneumococcal conjugate vaccine (PCV7) use in the pediatric community (61 months surveillance), after PCV13 implementation in children (100 months surveillance), and calculated as follows: VE = 1 -(cases per month with specific pneumococcal conjugate vaccination/cases per month without any pneumococcal conjugate vaccination).

Results

Following 15 years of active surveillance, we identified 21 cases of OM. At admission the median age of patients was 38 months (six months to 15 years old), the median hospitalization days was 12 (5 to 115). All patients underwent mastoidectomy. Identification of bacterial pathogens was possible in 19 (90.5%), among which. *Streptococcus pneumoniae *was the leading cause with 15 cases (79%). PCV7 VE was 27.8%, however, after PCV13 introduction, VE increased to 68%, with only one case of pneumococcal OM in the last two years, without incremental OM cases by other bacteriae.

Conclusion

After 15 years of active/prospective surveillance in our hospital, a continuous and high VE (68%) of PCV13 on pediatric OM caused by *Streptococcus pneumoniae* has been found, with only one case in the last two years.

## Introduction

Both the 7- and 13-valent pneumococcal conjugate vaccines (PCV-7 and PCV-13, respectively) have mostly been implemented and proved to be highly effective in decreasing several invasive pneumococcal diseases (IPD) in children (and adults by herd effect), including septicemia, meningitis, pleural empyema, among others [1-10]. Even though these vaccines initially were not implemented to reduce acute otitis media (AOM), various studies have shown its effects on decreasing this “mucosal” disease as a result of reducing both nasopharyngeal carriage and the first AOM episode when administered promptly in infancy [11-14].

However, its effectiveness in decreasing otomastoiditis (OM), a local/suppurative complication of AOM, has barely been reviewed [15-18]. We have performed several studies related to IPD, particularly by looking at the effectiveness of PCV13 on overall IPD and meningitis and pleural empyema [19-21].

This is a continuation of our active surveillance previously published [22], and is the first study in Latin America, by using active surveillance, examining the effectiveness of this vaccine on pneumococcal pediatric OM.

## Materials and methods

Active surveillance identifying patients < 16 years of age with OM admitted at the “Hospital General de Tijuana” was performed from October 1, 2005, to September 30, 2019. Diagnosis of OM was based on clinical (postauricular tenderness, erythema, and swelling causing protrusion of the auricle) and computerized tomographic signs (opacification of the mastoid air cells and middle ear). Bacterial isolation was obtained either from mastoids (in the operating room) and/or supra-mastoid abscesses (when present) and bacterial identification by conventional cultures or PCR.

For RT-PCR, DNA was extracted from the mastoid exudate and then processed. An RT-PCR assay (Stratagene®, USA) was used to identify six common pathogens that cause OM (*S.* *pneumoniae*, *Haemophilus influenzae*, *Moraxella catharralis*, *Escherichia coli, Streptococcus pyogenes*, and *Staphylococcus aureus*).

For *Streptococcus pneumoniae* isolates, serotyping was performed by the Quellung Reaction (Statens Serum Institute®, Copenhagen, Denmark), or by molecular serotyping using a sequential multiplex PCR method in which clinical isolates within each targeted serotype were amplified with its corresponding primer sets.

To assess pneumococcal conjugate vaccines effectiveness (VE), due to different periods of active surveillance between vaccines introductions and use, we did the following: we counted the number of cases per month (instead of the total number of cases) in different periods. Primarily, before any pneumococcal conjugate vaccine was implemented (19 months surveillance). Secondly, during the seven valent pneumococcal conjugate vaccine (PCV7) use in the pediatric community (61 months surveillance). And lastly, after PCV13 implementation in children (100 months surveillance).

Finally, we estimated VE by using the following formula: VE = 1- (cases per month with specific pneumococcal conjugate vaccination/cases per month without any pneumococcal conjugate vaccination).

Analysis of all information was merely descriptive using Excel®.

This study was approved by the Ethics Committee of the “Hospital General de Tijuana” (CONBIOETICA02CEI001), approval number 20170526.

## Results

Following 15 years of active surveillance, we identified 21 cases of OM. At admission, the median age of patients was 38 months (six months to 15 years old), and the median hospitalization days was 12 (5 to 115). All children had at least two doses of either PCV7 or PCV13 during both the PCV7 and PCV13 vaccination periods.

**Figure 1:**
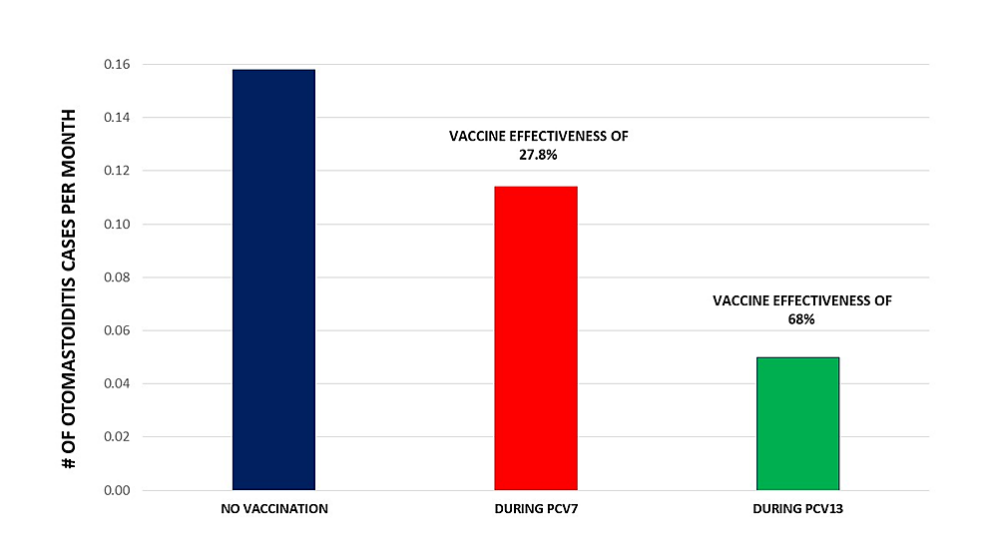
Otomastoiditis cases per month: before PCV7 (“No Vaccination”), during PCV7 universal vaccination (“During PCV7”), and during PCV13 universal vaccination (“During-PCV13”). PCV7: pneumococcal conjugate 7-valent vaccine; PCV13: pneumococcal conjugate 13-valent vaccine.

All patients underwent mastoidectomy. Identification of bacterial pathogens was possible in 19 (90.5%, 14 by culture, and five thru PCR), among which *Streptococcus pneumoniae* was the leading cause with 15 cases (79%), followed by two cases of *Streptococcus pyogenes*, and one case each of *Streptococcus anginosus *and *Proteus mirabilis*. PCV7 VE was 27.8% (0.158 cases per month prior to pneumococcal conjugate vaccination vs. 0.114 during PCV7 immunization) as previously published [22], however, after PCV13 introduction, VE increased to 68% (0.05 cases per month, see Figure 1).

Regarding serotype-specific PCV13 protection, only four pneumococcal OM cases were found during 100 months of surveillance, with only one case of a PCV13-related serotype (serotype 3), and four children had OM due to serotypes 33F, 35B, 24F, and 22F (one case each), with only one case of pneumococcal OM in the last two years. Additionally, PCV13 VE compared to PCV7 was 56% (0.05 vs. 0.114 cases per month). Furthermore, there were no incremental OM cases by other bacteriae.

## Discussion

As we previously stated in the Introduction section, both PCV7 and PCV13 were mostly implemented to prevent IPD, and not initially introduced to reduce acute otitis media (AOM). Nonetheless, particularly with PCV13, this added beneficial effect on AOM has been proved to be present in several studies [11-14]. Furthermore, a study performed in Israel by Lewnard, et al. [13], strongly suggested that early PCV13 vaccination in children avoids a high proportion of first episodes of AOM (in young infants most are caused by *Streptococcus pneumoniae*), preventing further development of a biofilm in the middle ear, therefore, in great proportion, impeding AOM due to other bacteriae, such as *Haemophilus influenzae *or *Moraxella catharralis*, as well as chronic middle ear effusion.

Nevertheless, publications particularly searching for the effect of either PCV7 and/or PCV13 on OM are scarce [15-18].

The Italian 15 years retrospective study done by Balsamo, et al. [15] did not show a statistical difference of OM before and after any PCV used, however, from all 143 patients enrolled in this publication, isolation was not reported, maybe because many patients did not undergo mastoidectomy and cultures not taken. On the other hand, in the study by Marom et al. [16], the authors retrospectively analyzed all causes by both uncomplicated and complicated AOM in the USA between 2001 and 2011. The results from this review did not show any changes of OM by PCV7, however, since 2009, following PCV13 implementation, attack rates of OM dropped from 0.6/100,000 to 0.32/100,000 (p = 0.05) strongly suggesting an effect of this vaccine on all causes of bacterial OM [16].

Similar findings are published by Tamir et al. [17], from which, based on their findings, PCV13 had more effect than PCV7 on Pneumococcal OM in Israel. Additionally, a study performed in Cincinnati (USA) by Tawfik, et al. [18] reported that PCV13 most likely was the cause of OM hospitalizations reduction in very young children (0-2 years old) between 2009-2012, however, both PCV7 and PCV13 did not have an overall effect on all ages in the interval between 2000-2012 [18].

As mentioned, there are no Latin American publications of VE of any PCV on pediatric OM. A systematic review by Valenzuela, et al. from 1990 to 2006 [23], estimates an annual burden of pneumonia, meningitis, and acute otitis media caused by pneumococcus in children < 5 years of age ranged from 980,000 to 1,500,000; 2,600 to 6,800; and 980,000 to 1,500,000; respectively, but no data on OM was analyzed.

When it comes to serotype-specific protection, it is relevant to mention the importance of higher invasiveness and antibacterial resistance of pneumococcal serotype 19A [1-4, 19, 20, 24, 25]. In Latin America, the study performed by Avila-Aguero, et al. [24] showed that in countries where 10-valent pneumococcal conjugate vaccine (PCV10) has been implemented, IPD due to serotype 19A (present in PCV13, but not in PCV10), has raised; while in countries where only PCV13 is being used serotype 19A is decreasing, yet this study looked neither at AOM and/or OM.

Nonetheless, a publication by Kaplan, et al. [25], over a three-year period (2011-2013) in 8 pediatric hospitals in the USA, the number of pneumococcal serotype 19A strains isolated from either AOM or OM decreased by 76% since PCV13 introduction, also leading to less antibacterial resistance. In our previous published study [22], during 61 months of surveillance, while PCV7 was routinely administered as part of the National immunization program, there were seven cases of pneumococcal OM (0.114 per month), six of them serotypes included in PCV13 (but not in PCV7), and three patients developed OM by serotype 19A, one who also developed meningitis and permanent lesions to cervical vertebrae. As previously shown in the Results section, following PCV13 vaccination, only one PCV13 serotype-related case was seen, with no cases of serotype 19A.

We acknowledge that our study comes from data done only in one hospital; nonetheless, it is based on active/prospective surveillance for a long period (15 years), and yearly updated. Mastoidectomy was performed in all patients, and bacterial recovery was successful in 90% of patients. Additionally, following the studies done in the USA [16,25], and to our previous publication [22], we also did not see an important impact by PCV7 on AOM, yet, it was higher with PCV13 (68%), due to a higher vaccine serotype coverage, including serotypes causing OM. In addition, in our study, we only prospectively looked at pneumococcal confirmed OM by either PCR or culture, and PCV13 has proven continuous effectiveness.

## Conclusions

In this 15-year active surveillance study, a follow-up and complement of the first active/prospective study searching for OM in Latin American children, *Streptococcus pneumoniae* was the leading cause of OM. Additionally, continuous effectiveness after the implementation of PCV13 is present, leading to almost 100% pneumococcal PCV13 serotype-specific, and 68% all pneumococcal serotypes reductions, respectively, in addition to no emergence of other bacteriae. In addition, in order to reinforce PCV13 effectiveness on OM, a merely mucosal complication of AOM, further active surveillance needs to be developed in other hospital settings, as well as other studies, mainly the ones looking at the nasopharyngeal carriage.
